# Sarcopenia and associated risk factors in oncology outpatients in specialized cancer centers in Saudi Arabia: a cross-sectional study

**DOI:** 10.1097/MS9.0000000000000794

**Published:** 2023-05-08

**Authors:** Nabil Almouaalamy, Sittelbenat H. Adem, Abdulrahman A. Alsubhi, Ahmed B. Alansari, Marwan A. Yahya, Sultan A. Alsadan

**Affiliations:** a Oncology Department, Princess Noorah Oncology Center; b Nursing Department, King Abdulaziz Medical City, Ministry of National Guard-Health Affairs; c College of Medicine, King Saud bin Abdulaziz University for Health Sciences; d King Abdullah International Medical Research Centre, Jeddah, Saudi Arabia

**Keywords:** cancer, muscular atrophy, sarcopenia, screening, secondary prevention

## Abstract

Sarcopenia is a comorbidity associated with cancer, which takes advantage of a patient’s deteriorating state and decreases the survival rate. Thus, early detection of sarcopenia could improve oncology outpatient prognosis, treatment, and general quality of life.

**Objective:**

To measure the prevalence of probable sarcopenia and the associated risk factors in patients with cancer.

**Methods:**

This is a cross-sectional study. A total of 324 cancer patients were screened for sarcopenia using a simple questionnaire to rapidly diagnose sarcopenia [SARC-F (strength, assistance with walking, rising from a chair, climbing stairs, and falls)] and a hand grip dynamometer tool. The study was conducted from 1 January 2021 till 28 February 2021, in the outpatient department of Princess Noorah Oncology Center.

**Results:**

Among 324 cancer patients receiving active chemotherapy treatment, 28.4% screened positive for sarcopenia (SARC-F score ≥4). Moreover, 23.45% were identified as probable cases [SARC-F score ≥4 and a low hand grip strength (HGS)] of sarcopenia, according to the European Working Group on Sarcopenia in Older People consensus 2 (EWGSOP2) case-finding algorithm, which is sufficient to initiate a management plan.

**Conclusions:**

One-quarter of the cancer patients had probable sarcopenia at our institution. Sarcopenia risk was independently associated with patient age, and the risk of sarcopenia was low among patients with higher albumin concentrations. Screening cancer patients for sarcopenia using the SARC-F questionnaire and HGS may offer a useful strategy to mitigate the risk of unfavorable consequences that may occur during cancer treatment.

## Background

Highlights

- One-quarter of the cancer patients had probable sarcopenia at our institution.
- Sarcopenia risk was independently associated with advanced age, and the risk was reduced with higher serum albumin concentrations.
- Screening cancer patients for sarcopenia using the SARC-F (strength, assistance with walking, rising from a chair, climbing stairs, and falls) questionnaire and hand grip strength (HGS) may offer a useful strategy to mitigate the risk of unfavorable consequences that may occur during cancer treatment.
- Patients with established sarcopenia may benefit from a multimodal approach comprising physical therapy and nutritional supplementation.

Sarcopenia is a condition characterized by the degradation and weakening of skeletal muscles, eventually resulting in loss of function^1^. Sarcopenia is widely associated with the elderly population, but this condition is also associated with risk factors other than age, such as sex, chemotherapy, nutrition, and physical activity level^1^. The prevalence of sarcopenia varies depending on the population, from 5–13% among elders aged 60–70 years to 11–50% among those aged at least 80 years^2^. The most common cancer diagnoses in Saudi Arabia were breast cancer (14.8%), colorectal cancer (14.6%), and thyroid cancer (10.1%)^3^.

Due to intrusive treatments for cancer, such as surgery, chemotherapy, and radiation, sarcopenia is considered one of the many comorbidities of cancer, as it takes advantage of the patient’s deteriorating state and decreases the survival rate^4,5^. Thus, early detection of sarcopenia could improve oncology outpatients’ prognosis, treatment, and general quality of life, not enough to reverse the effects of sarcopenia but sufficient to halt its progress^6–8^. In clinical practice, a simple questionnaire to rapidly diagnose sarcopenia [SARC-F (strength, assistance with walking, rising from a chair, climbing stairs, and falls)] is used to screen the risk of sarcopenia among oncology patients. The SARC-F is a quick, simple, and easy-to-use questionnaire containing five items^9^. It has been tested in different clinical settings^10^ and against multiple gold standard diagnostic methods^11,12^. SARC-F has a low sensitivity of 35.3% and a high specificity of 85.7%^11^. In 2018, SARC-F was incorporated as a sarcopenia case-finding tool in the European Working Group on Sarcopenia in Older People consensus 2 (EWGSOP2) diagnostic algorithm for sarcopenia^13^. The goal of this study was to measure sarcopenia in cancer patients as well as the risks associated with these patients.

## Methods

This study is a cross-sectional study that investigated sarcopenia and its associated risk factors among oncology patients. The study was conducted from 1 January 2021 till 28 February 2021, in the outpatient department of Princess Noorah Oncology Center (PNOC). The PNOC is a tertiary cancer center with inpatient and outpatient departments. This article has been reported in line with the STROCSS criteria^14^.

### Patient selection

From a population of 2000 patients, the sample size was calculated using Raosoft software with a confidence level of 95% and 5% margin of error, resulting in the selection of 323 patients. However, we included 324 patients. All study participants provided written informed consent, and the study was conducted according to the guidelines of the Declaration of Helsinki and approved by King Abdullah International Medical Research Center Research Centre ethics review board, study number SP20/061/J on 1 June 2020. The study included patients aged more than 18 years, had cancer, and were on chemotherapy. Patients who had privacy requests, did not meet the inclusion criteria, or had a physical deformity that made it impossible for them to be tested for muscle strength were excluded. Convenience sampling was used to recruit patients in the study.

### Hand grip strength

Hand grip strength (HGS) was assessed in our study using the Jamar PLUS + Hydraulic Hand Dynamometer (Patterson Medical Cedarburg, Wisconsin, USA) set at the second handle position because of its suitability and reliability^15^. We adopted the American Society of Hand Therapists (ASHT) protocol^16^. A healthcare provider provided verbal instructions and demonstrated each testing procedure before the assessments were conducted. The scores were recorded for three successive trials for the dominant hand with a 2-min rest period between each trial.

### A simple questionnaire to rapidly diagnose sarcopenia (SARC-F)

The questionnaire used in this study was SARC-F in English, which is indicated for screening the risk of sarcopenia. It is composed of five objective questions and is self-reported by the patient in a direct interview. A bilingually trained healthcare provider conducted interviews with the patients.

### Thresholds and case-finding of sarcopenia

Sarcopenia was diagnosed according to the EWGSOP2^12^. The sex-standardized grip strength cut-off values were 70.5 lbs (32 kg) for men and 48.5 lbs (22 kg) for women based on values from regional studies^17,18^. We used the SARC-F questionnaire to identify sarcopenia cases and applied HGS to identify probable sarcopenia cases, as per the EWGSOP2 algorithm for case-finding.

### Statistical data analysis

Mean and standard deviation (SD) statistics were used to describe continuous variables (age, weight, height, body mass index (BMI), serum albumin, serum hemoglobin, treatment cycle number, grip strength measurements, and SARC-F total score), and frequencies and percentages were used for categorical variables (gender, age group, marital status, BMI categories, city of residence, type of cancer, type of chemotherapy, dominant hand, frequencies of normalized HGS, and SARC-F score category). Cronbach’s alpha was applied to the five indicators of the SARC-F. In addition, the normality assumption for continuous variables was tested statistically using the Kolmogorov–Smirnov test of normality, which was non-normally distributed *P*<0.0001, and visually using a histogram.

The dichotomy analysis was applied to describe the multiple-response variables, and Pearson’s correlation test was used to assess the correlations between the SARC-F score and other measured continuous variables and outcomes. The independent sample *t*-test was used to compare the continuous measured variables across the levels of dichotomous variables for statistically significant differences, and the *χ* ^2^ test of independence was used to assess the associations between categorical variables. Multivariable binary logistic regression analysis was used to regress the patients’ odds of having sarcopenia against their sociodemographic and clinical characteristics. The associations between these factors and the patients’ odds of sarcopenia were expressed as odds ratios (OR) with associated 95% confidence intervals (CI). The IBM commercially available statistical software SPSS version 22 was used for data analysis, and the alpha significance level was set at 0.050.

## Results

### Demographic and clinical characteristics

**Figure 1:**
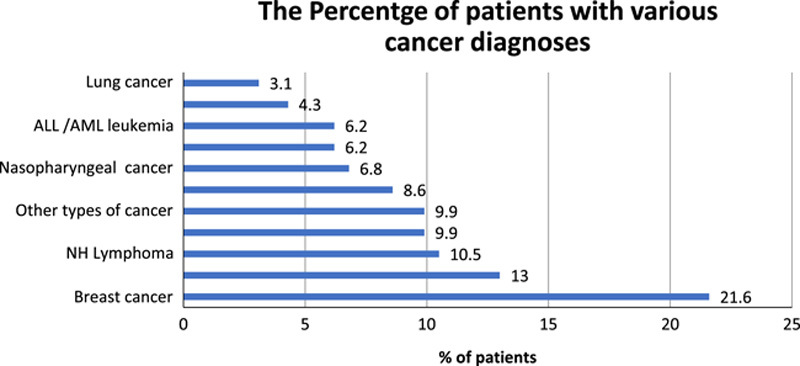
The percentage of patients with various cancer diagnoses. ALL, acute lymphocytic leukemia; AML, acute myeloid leukemia; NH, non-Hodgkin’s.

In total, 324 patients were included in the present study. More than half of the patients were female (53.1%), resided in Jeddah (52.5%), and were married (84%). Patients had a mean age of 51.8±15.8 years and a BMI of 27.8±6.5 kg/m^2^. The remaining demographic characteristics are shown in Table 1. Regarding clinical characteristics, the most predominant types of cancer were female breast cancer (21.6%), colorectal cancer (13.0%), and non-Hodgkin’s lymphoma (10.5%, Fig. 1). Most patients received curative treatment (98.1%), whereas only 1.9% received palliative treatment. Carboplatin (20.4%) and doxorubicin (16.7%) were the most frequently used chemotherapeutic agents. Detailed descriptive data regarding clinical characteristics are listed in Table 1.

**Table 1:** Descriptive analysis of the cancer-diagnosed patients’ sociodemographic characteristics, *N*=324

	Frequency	Percentage
Sex		
Female	172	53.1
Male	152	46.9
Age (years), mean (SD)		
Age group		
≤30	36	11.1
31–40	44	13.6
41–50	66	20.4
≥51	178	54.9
Marital status		
Never married	52	16
Ever married	272	84
Weight (kg), mean (SD)		72.64 (18.96)
Height (cm), mean (SD)		161.50 (10.46)
BMI, mean (SD)		27.82 (6.50)
BMI classification		
Underweight	26	8
Normal	82	25.3
Overweight	104	32.1
Obese class I	46	14.2
Obese class II	54	16.7
Obese class III	12	3.7
Residence		
Makkah	50	15.4
Jeddah	170	52.5
Almadina	56	17.3
Al Taef	20	6.2
Assir	4	1.2
Albaha	14	4.3
Jazan	6	1.9
Tabuk	2	0.6
Alqaseem	2	0.6

BMI, body mass index; SD, standard deviation.

### Descriptive data of HGS and the results of SARC-F responses

Among all patients, 63.6% were right-handed and 36.4% were left-handed. The overall mean and grip strength (HGS) score for the three HGS measures combined was 42.1±16.4 lbs. Abnormal sex-standardized HGS was reported in approximately two-thirds of the patients (66.0%). Regarding the self-reported results of the SARC-F questionnaire, the mean±SD SARC-F score was 1.8±2.0, with 28.4% of patients having a score of at least 4 points. The most frequent subdomains in which the patients experienced much difficulty included the strength subdomain (11.1%), followed by climbing stairs (9.9%), and falls (5.6%) (Table 2).

**Table 2:** Descriptive analysis of the cancer patients’ measured sarcopenia SARC-F and strength indicators of difficulty

	Frequency	Percentage
Dominant hand		
Left	118	36.4
Right	206	63.6
Grip strength measure #1 (lbs), mean (SD)		46.21 (17.27)
Grip strength measure #2 (lbs), mean (SD)		41.88 (16.51)
Grip strength measure #3 (lbs), mean (SD)		38.20 (16.21)
Overall mean grip strength (lbs), mean (SD)		42.10 (16.40)
HGS (lbs) standardized for patient sex		
Normal for sex	110	34
Abnormal for sex	214	66
SARC-F total score		1.80 (2.02)
SARC-F score dichotomized		
SARC-F score ≤4 points	232	71.6
SARC-F score ≥4 points	92	28.4
SARC-F sub-indicators		
Strength score		
No difficulty	184	56.8
Some difficulty	104	32.1
Much difficulty	36	11.1
Assistance in walking score		
No difficulty	226	69.8
Some difficulty	96	29.6
Much difficulty	2	0.6
Rise from a chair score		
No difficulty	250	77.2
Some difficulty	74	22.8
Much difficulty	0	0
Climb stairs score		
No difficulty	194	59.9
Some difficulty	98	30.2
Much difficulty	32	9.9
Fall score		
No difficulty	278	85.8
Some difficulty	28	8.6
Many falls	18	5.6

SARC-F, strength, assistance with walking, rising from a chair, climbing stairs, and falls; SD, standard deviation.

### The association among HGS, SARC-F, and other numerical variables

Correlation analyses between continuous variables showed significant positive associations between HGS and patient height (*r*=0.419, *P*<0.01), serum albumin concentrations (*r*=0.213, *P*<0.01), and hemoglobin levels (*r*=0.376, *P*<0.01). Furthermore, the SARC-F score was positively correlated with patient age (*r*=0.274, *P*<0.01) and negatively correlated with overall HGS score (*r*=0.450, *P*<0.01), patient height (*r*=0.253, *P*<0.01), serum albumin levels (*r*=0.301, *P*<0.01), and hemoglobin levels (*r*=0.200, *P*<0.05) (Table 3).

**Table 3:** Bivariate Pearson’s correlation between patients measured SARC-F score and other clinical outcomes and measures

	SARC-F score	Age (years)	Grip strength	Height	Weight	BMI	Albumin
Age (years)	0.274^*^						
Overall mean grip strength (lbs)	−0.450^*^	−0.199^†^					
Height (cm)	−0.253^*^	−0.151	0.607^*^				
Weight (kg)	−0.128	−0.137	0.370^*^	0.419^*^			
BMI	0.008	−0.048	0.075	−0.085	0.858^*^		
Serum albumin level	−0.301^*^	−0.449^*^	0.307^*^	0.213^*^	0.304^*^	0.172^†^	
Serum hemoglobin level	−0.200^†^	−0.056	0.376^*^	0.359^*^	0.355^*^	0.167^†^	0.383^*^

* Correlation is significant at the 0.01 level (two-tailed).

† Correlation is significant at the 0.05 level (two-tailed).

BMI, body mass index; SARC-F, strength, assistance with walking, rising from a chair, climbing stairs, and falls.

### Factors associated with risk of sarcopenia

Upon the dichotomization of the SARC-F score to identify patients with risk of sarcopenia, the results of the statistical differences showed that patients with risk of sarcopenia were significantly older (57.4±16.4 years vs. 49.6±15.0 years; *P*=0.004), had shorter heights (158.9±10.8 cm vs. 162.5±10.2 cm; *P*=0.047), had lower albumin concentrations (38.4±3.9 g/l vs. 40.6±4.1 g/l; *P*=0.002), and had lower overall HGS (33.3±13.7 cm vs. 45.6±16.1 cm; *P*<0.001) than those without risk of sarcopenia. In addition, significantly higher proportions of patients with risk of sarcopenia had abnormal sex-standardized HGS (82.6% vs. 59.5%, *P*=0.005) and used paclitaxel more frequently than patients without risk of sarcopenia (23.9% vs. 10.3%, *P*=0.026) (Table 4).

**Table 4:** Bivariate analysis of the patients with sarcopenia

			Sarcopenia (SARC-F score ≥4 points)		
			No (*n*=232)	Yes (*n*=92)	*P*
Sex					
Female			116 (50)	56 (60.9)	0.211
Male			116 (50)	36 (39.1)	
Age (years), mean (SD)			49.56 (15.04)	57.41 (16.40)	0.004
Marital status			
Never married			34 (14.7)	18 (19.6)	0.443
Ever married			198 (85.3)	74 (80.4)	
Weight (kilograms), mean (SD)			72.78 (19.30)	72.28 (18.30)	0.882
Height (centimeters), mean (SD)			162.48 (10.18)	158.87 (10.81)	0.047
BMI, mean (SD)			27.51 (6.40)	28.61 (6.72)	0.332
Type of treatment			
Curative			228 (98.3)	90 (97.8)	1
Palliative			4 (1.7)	2 (2.2)	
Treatment cycle number, median (Quartile 1, Quartile 3)			6.61 (7.71)	8.90 (13.45)	0.178
Serum albumin level, grams/l, mean (SD)			40.58 (4.10)	38.37 (3.88)	0.002
Serum hemoglobin level, grams/l, mean (SD)			11.73 (2.03)	11.28 (1.99)	0.202
Dominant hand			
Left			86 (37.1)	32 (34.8)	0.785
Right			146 (62.9)	60 (65.2)	
Overall mean grip strength (lbs), mean (SD)			45.60 (16.10)	33.29 (13.70)	<0.001
Sex-standardized grip strengtha			
Normal for sex			94 (40.5)	16 (17.4)	0.005
Abnormal for sex			138 (59.5)	76 (82.6)	
Use of paclitaxela			
No			208 (89.7)	70 (76.1)	0.026
Yes			24 (10.3)	22 (23.9)	

Note: None of the cancer diagnoses and the other chemotherapeutic agents correlated significantly with sarcopenia.

BMI, body mass index; SARC-F, strength, assistance with walking, rising from a chair, climbing stairs, and falls; SD, standard deviation.

To further corroborate the adjusted associations between risk of sarcopenia and different variables, we included risk of sarcopenia status as a dependent variable and significant demographic and clinical variables as independent variables. The results showed that risk of sarcopenia was independently associated with age [adjusted odds ratio (aOR)=1.06; 95% CI: 1.02–1.09; *P*=0.003], and abnormal sex-standardized HGS (aOR=3.61; 95% CI: 1.28–10.17; *P*=0.015). Married patients (aOR=0.12; 95% CI: 0.03–0.49; *P*=0.003) and those with higher albumin concentrations (aOR=0.89; 95% CI: 0.80–0.99; *P*=0.046) were less likely to have increased risk of sarcopenia (Table 5).

**Table 5:** Multivariate logistic binary regression analysis of the cancer patients’ odds of sarcopenia

		95% CI for OR		
	Multivariate-adjusted odds ratio	Lower	Upper	*P*
Sex (male)	0.894	0.375	2.130	0.801
Age (years)	1.055	1.018	1.093	0.003
Marital status (ever married)	0.118	0.029	0.491	0.003
Serum albumin level	0.894	0.802	0.998	0.046
Receiving paclitaxel chemotherapy	2.927	0.974	8.791	0.056
Sex-standardized HGS (abnormal for sex)	3.607	1.279	10.167	0.015
Constant	0.500			0.794

DV=SARC-F score ≥4 points (no/yes). The overall statistical significance of the model was set at *P*<0.001.

CI, confidence interval; HGS, hand grip strength; OR, odds ratio.

## Discussion

In this study, we examined the prevalence and risk factors of probable sarcopenia in patients with cancer attending the the outpatient department in PNOC. Among 324 patients with cancer on active chemotherapy, 28.4% screened positive for sarcopenia (SARC-F score ≥4). Moreover, 23.45% were identified as probable cases (SARC-F score ≥4 and a low HGS) of sarcopenia as per the EWGSOP2^12^ case-finding algorithm, which is clinically sufficient to initiate a management plan. Advanced age (>57 years) was independently associated with probable sarcopenia, which is similar to the findings of other studies^19,20^. In addition, being married and having an adequate albumin concentration were associated with a decreased risk of sarcopenia in the study sample.

In the literature, the prevalence of sarcopenia among patients with cancer varies considerably according to the type of cancer and disease stage, and it relies on the tools used for quantifying the disease. For instance, the prevalence of sarcopenia ranges from 12 to 60% among patients with colorectal cancer^10,21–23^, 15.9 and 22.4% among patients with breast cancer^24,25^, 52% among patients with small cell lung cancer, and 43% among patients with non-small cell lung cancer^26^. Intriguingly, we showed that patients with probable sarcopenia had significantly lower serum albumin levels than their peers without sarcopenia. This was in agreement with a recent cross-sectional study^27^ in which circulating albumin levels were significantly lower among patients with sarcopenia and severe sarcopenia than in those without sarcopenia. Furthermore, albumin was deemed a predictive biomarker for sarcopenia, with an optimal cut-off point of less than 41.3 g/l, sensitivity of 71.7%, and specificity of 66.7%^27^.

In the present study, age was positively associated with probable sarcopenia in both univariate and adjusted multivariate analyses. In otherwise normal adults aged more than 50 years, muscle strength has been shown to be significantly reduced at a rate of 12–14% per decade, and this is apparently associated with decreased muscle mass^28–30^. Multiple factors could contribute to age-related sarcopenia, such as hormonal and lifestyle factors as well as age-dependent biological changes. Interestingly, sarcopenia may be further triggered by a response to specific medications, such as steroids and chemotherapeutic medications, which may partly explain the high prevalence of sarcopenia among patients with cancer. Furthermore, while physical inactivity is associated with the loss of lean body mass, chemotherapy is linked to a marked increase in fat mass, which causes a marked shift in the lean body/fat mass ratio^31,32^.

In our study, we used the revised EWGSOP2^12^ to establish the diagnostic criteria of the disease based on HGS as the primary outcome because muscle strength is inherently the most reliable measure of muscle function. The EWGSOP2 expert panel relied on normative data retrieved from 12 British studies^33^. Nevertheless, it has been recommended that the appropriate cut-off values should be exclusively based on regional or national normative data rather than data from European populations due to variations in stature. Therefore, future national guidelines in Saudi Arabia are warranted, considering the use of national reliable data to set HGS cut-off values for the Saudi population.

In our study, the fact that one-quarter of the patients had probable sarcopenia underscores the importance of implementing urgent procedures in the management of cancer patients. A multimodal approach comprising exercise programs and adequate nutritional support may be necessary to preserve the muscular mass^23^. Furthermore, the early detection of sarcopenia once cancer has been diagnosed seems to be protective against the unfavorable consequences that may develop during subsequent chemotherapeutic, radiotherapeutic, and surgical interventions. However, the efficacy of such interventions seems to be influenced by pre-treatment body composition, cancer stage at diagnosis, and the receipt of anticancer interventions. Collectively, it is recommended to add sarcopenia screening to the predefined clinicopathological scoring for cancer patients prior to oncological treatment. Our study was limited by the cross-sectional nature of the correlations between sarcopenia and HGS, and other variables could not exclusively indicate causal inferences or directionality. In addition, the patients in our study were recruited from a single institution, which may limit the generalizability of our findings across other national, regional, and international cohorts.

## Conclusions

In conclusion, one-quarter of the cancer patients had probable sarcopenia at our institution. Sarcopenia risk was independently associated with advanced age, and the risk was reduced with higher serum albumin concentrations. Screening cancer patients for sarcopenia using the SARC-F questionnaire and HGS may offer a useful strategy to mitigate the risk of unfavorable consequences that may occur during cancer treatment. Additionally, patients with established sarcopenia may benefit from a multimodal approach comprising physical therapy and nutritional supplementation. Further studies with larger sample sizes and more interventional tools are required to confirm our findings.

## Ethical approval

The study was conducted according to the guidelines of the Declaration of Helsinki and approved by King Abdullah International Medical Research Centre ethics review board, study number SP20/061/J on 1 June 2020.

## Consent

Informed verbal and written consent was obtained from all subjects involved in the study.

## Sources of funding

This research received no specific grant from any funding agency in the public, commercial, or not-for-profit sectors.

## Author contribution

N.A.: conceptualization; N.A.: methodology; N.A. and S.H.A.: software; N.A. and S.H.A.: validation; S.H.A.: formal analysis; S.H.A., A.A.A., A.B.A., M.A.Y., and S.A.A: investigation; S.H.A. and N.A.: resources; N.A., S.H.A., A.A.A., A.B.A., M.A.Y., and S.A.A.: data curation; N.A.: writing – original draft preparation; N.A., S.H.A., A.A.A., A.B.A., M.A.Y., and S.A.A.: writing – review and editing; N.A., S.H.A., A.A.A., A.B.A., M.A.Y., and S.A.A.: visualization; N.A.: supervision; N.A.: project administration; N.A., S.H.A., A.A.A., A.B.A., M.A.Y., and S.A.A.: funding acquisition. All authors have read and agreed to the published version of the manuscript.

## Conflicts of interest disclosure

The authors declare that there are no conflicts of interest.

## Research registration unique identifying number (UIN)

Name of the registry: not applicable.
Unique identifying number or registration ID: 8816.
Hyperlink to your specific registration (must be publicly accessible and will be checked): not applicable.

## Guarantor

Nabil Almouaalamy, Consultant Geriatric & Palliative Medicine, Princess Noorah Oncology Center, King Abdulaziz Medical City, Ministry of National Guard-Health Affairs, Makkah/Jeddah Highway, Jeddah 21423, Saudi Arabia. Tel: +966 55 556 1560, E-mail: mouaalamyn@ngha.med.sa

## Data availability statement

The study data were obtained from subjects, available with the principal investigator, and available as per Data Availability Statements.

## Peer review

Not commissioned, externally peer-reviewed.
